# Optimization of ethanol-extracted lignin from palm fiber by response surface methodology and preparation of activated carbon fiber for dehumidification

**DOI:** 10.1186/s40643-022-00549-9

**Published:** 2022-05-30

**Authors:** Jie Fan, Qiongfen Yu, Ming Li, Jie Chen, Yunfeng Wang, Ying Zhang, Guoliang Li, Xun Ma, Hao Zhong, Yamei Yu

**Affiliations:** 1 Solar Energy Research Institute, Yunnan Normal University, Kunming, 650500 China; 2 Key Laboratory of Solar Heating and Cooling Technology of Yunnan Provincial Universities, Kunming, 650500 China

**Keywords:** Palm fiber, Lignin extraction, Organic solvent, Response surface methodology, Solvent recycling, Lignin-based activated carbon fiber, Water vapor adsorption

## Abstract

Lignin is a renewable bioresource that can be used for a variety of value-added applications. However, the effective separation of lignin from lignocellulosic biomass remains an ongoing challenge. In this study, lignin was extracted from waste palm fiber and successfully converted into a dehumidifying material. The following four process parameters of lignin extraction from palm fiber were optimized systematically and comprehensively using the response surface methodology: reaction time, extraction temperature, ethanol concentration and solid/liquid ratio. The results revealed that under the optimum processing conditions (111 min of extraction at 174 °C using 73% ethanol at 1/16 g/mL solid/liquid ratio), the extraction yield of lignin was 56.2%. The recovery of ethanol solvent was as high as 91.8%. Further, the lignin could be directly used without purification to produce lignin-based activated carbon fibers (LACFs) with specific surface area and total pore volume of 1375 m^2^/g and 0.881 cm^3^/g, respectively. Compared with the commercial pitch-based activated carbon fiber, the LACF has a higher specific area and superior pore structure parameters. This work provides a feasible route for extracting lignin from natural palm fiber and demonstrates its use in the preparation of activated carbon fiber with a remarkable performance as a solid dehumidification agent.

**Graphical Abstract:**

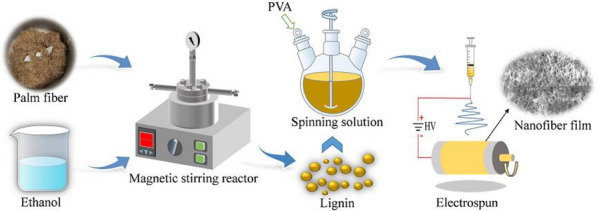

## Introduction

With the development of economy and the widespread use of fossil fuels, greenhouse gas emissions have been increasing (Yaqoob et al. 2020), resulting in the shortage of fossil energy and environmental pollution (Cai et al. 2021). It is therefore important to find renewable energy sources for resolving the energy crisis and environmental issues (United Nations 2020). Lignocellulosic biomass, which occurs widely in nature and is also inexpensive, can be considered a renewable and clean energy resource (Arora et al. 2015). Lignin is a natural organic polymer synthesized during the secondary metabolism of plants, and together with cellulose and hemicellulose, it constitutes one of the main components of the plant skeleton. Process optimization is required for the effective extraction of lignin for industrial applications (Florian et al. 2019). Palm fiber, as a natural fiber, is widely distributed around the world and is an important renewable resource (Chen et al. 2018). Moreover, palm fiber has a variety of reactive groups and a higher lignin content than flax and bamboo (Wang et al. 2016). However, palm fiber is discarded as waste and most of it is disposed of by burning or landfilling, resulting in considerable pollution and loss of resource. Therefore, if natural palm fiber is used as a source of lignin, a high-value utilization of palm fiber can be achieved to realize environmental and economic benefits.

Lignin samples differ considerably in structure and physicochemical properties owing to different natural sources and different methods used for extraction. Therefore, the separation of lignin from lignocellulosic materials is one of the most challenging and key processes in biological refining. Currently, sulfate lignin, soda lignin, and Kraft lignin are the main commercially available lignin samples (Huang et al. 2020). However, the high sulfur and ash content of sulfate lignin is not conducive to its further processing and application (Evdokimov et al. 2018). Although lignin obtained by alkali pulping does not contain sulfur, the extraction method has certain limitations, such as a low extraction yield, high alkali consumption and environmental pollution (Xu et al. 2020). The purity of lignin extracted by conventional methods is not high, which limits its further processing. Consequently, organic solvent-based extraction methods are being developed to ensure that the extracted material has similar characteristics to those of natural lignin (Paulsen Thoresen et al. 2021). A survey of literature on lignin extraction reveals that the extraction yield and structural properties of lignin obtained via organic solvent-based extraction are significantly affected by the extraction parameters such as the reaction time, extraction temperature, solvent concentration and solid/liquid ratio (Asadi and Zilouei 2017). Therefore, the parameters of lignin extraction need to be optimized to take full advantage of the organic solvent method.

The main factors that affect the separation of lignin by the organic solvent method are complex. Therefore, it becomes particularly important to explore the interaction between the lignin extraction rate and various operational parameters. In this study, we used the response surface methodology (RSM) to evaluate the interaction between several parameters such as the reaction time, extraction temperature, ethanol concentration, and solid-to-liquid ratio, and thereby optimize the processing parameters using a regression model to determine the optimal extraction conditions. The RSM has previously been successfully applied to optimize the parameters of lignin extraction from wheat straw (time, temperature, and pressure) (Ramezani and Sain 2018), oil palm biomass (temperature, time, and solid loading) (Rashid et al. 2018), rice straw (biomass loading, surfactant concentration, and time) (Sindhu et al. 2012) and sugarcane bagasse (NaOH concentration, time, and solid–liquid ratio) (Terán Hilares et al. 2016). However, in previous studies, only the effects of three processing parameters on the lignin extraction yield were often explored, and there is a lack of studies on a more comprehensive optimization of the processing conditions. One of the recent studies focused on the extraction of lignin from empty fruit bunch and palm kernel shell residues produced by the palm oil industry (Rashid et al. 2018). However, studies on the direct extraction of lignin from natural palm fiber are scarce. Moreover, although Rashid et al. (2021) successfully extracted lignin from oil palm biomass, they did not demonstrate the application value of the extracted lignin. Most of the current studies on the preparation of lignin-based carbon fibers (LCFs) are based on commercial alkali lignin or sulfate lignin, which often require further purification or modification before use. In addition, the previous studies were generally focused on the development of capacitor electrode materials (Hu et al. 2022) and adsorbents for volatile organic compounds (Song et al. 2019), and there are no reports on LCFs as dehumidifiers.

Therefore, in this study, the RSM was used to more comprehensively and systematically examine the effects of four processing parameters, viz., the reaction time, extraction temperature, ethanol concentration, and solid/liquid ratio on the lignin extraction yield. The thermal stability, surface functional groups, molecular weight, and structural characteristics of the extracted lignin were also analyzed. Finally, to explore the utilization value of the extracted palm fiber-based lignin, the feasibility of preparing LCFs by electrostatic spinning and the water vapor adsorption capacity of the lignin-derived carbon fibers were investigated to explore their potential as solid dehumidification materials.

## Materials and methods

### Materials and instruments

The palm fibers used in this study were obtained from a local plantation in Azahe Township, Honghe County, Honghe Prefecture, Yunnan Province, China. Sodium hydroxide, sulfuric acid (98% assay), ammonium oxalate, anhydrous ethanol, barium chloride, *N*,*N*-dimethylformamide (DMF), and polyvinyl alcohol (PVA) were obtained from Shanghai Aladdin Reagent Co. All these chemicals were of analytical reagent grade. The micromagnetic stirring reactor (TGYF-550/500-2J) used in this study was purchased from Chengdu Xingtianyu Experiment Apparatus Co., Ltd. The main technical parameters of the reactor are as follows: maximum pressure, 15 MPa; temperature stability, ≤ ± 1 ℃, and stirring rate, 0–1400 r/min.

### Determination of the main chemical components of palm fiber

The chemical composition of the palm fiber used in this study was determined based on the “Quantitative Analysis Method of Ramie Chemical Composition” (GB/T 5889-1986) and the analysis approaches established by the National Renewable Energy Laboratory (NREL) (Sluiter et al. 2008). The contents of fat wax, water-soluble compounds, pectin, hemicellulose, cellulose, and Klason lignin were determined, and the specific test methods are listed in Table 3. All experiments were run in triplicates.

### Extraction of lignin from palm fibers

#### Pretreatment

Palm fiber sheets were soaked in water for 24 h and then gently rubbed to remove dust and impurities. The palm fiber bundles were then cleaned with distilled water and desiccated in an oven at 105 °C for 4 h. Subsequently, the dried palm fibers were crushed and passed through a 60 mesh sieve. The palm fiber powder was treated with boiling water for 2 h and then washed several times with cold water. Finally, the palm fiber powder was dried thoroughly in an oven at 60 °C for 12 h.

#### Organic solvent extraction of lignin

**Fig. 1:**
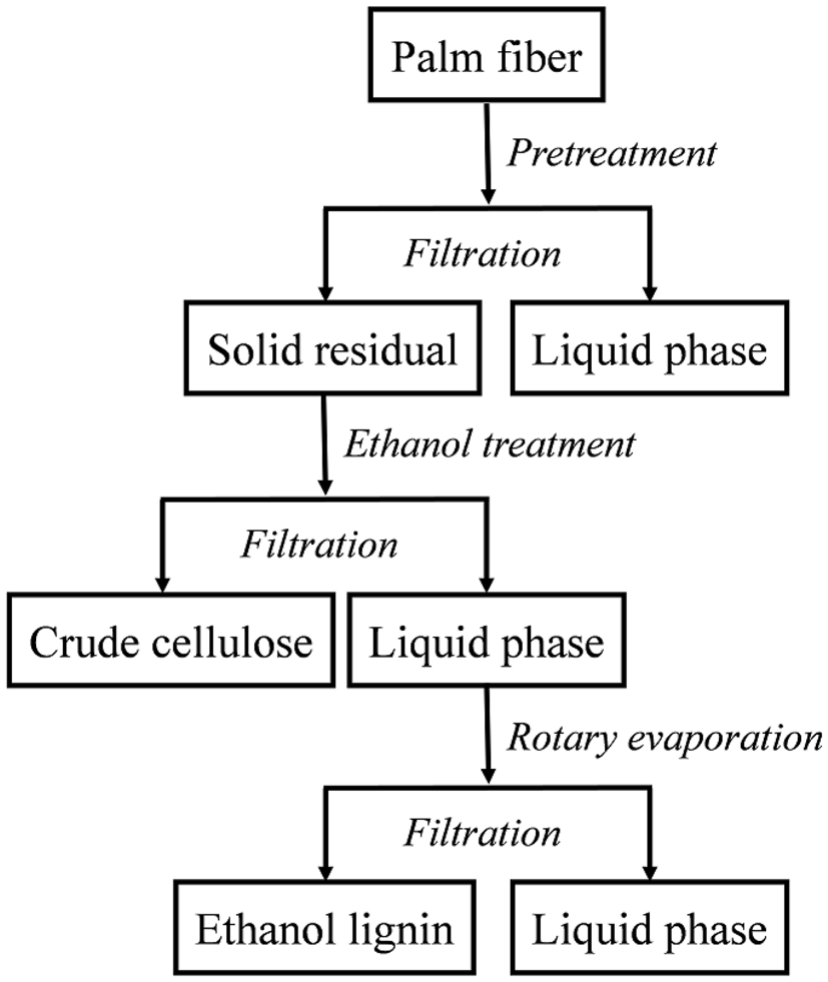
Flow diagram of the separation of lignin from palm fibers via ethanol extraction

The multi-step extraction process of lignin from the palm fiber powder is illustrated in Fig. 1. The organic solvent extraction was performed in a miniature magnetically stirred reactor with a volume capacity of 500 mL. The extraction solvent consisted of ethanol, water, and sulfuric acid at a volume ratio of 70:28:2. The dried palm fiber powder (8.0 g) and extraction solvent were mixed in the reactor at a solid/liquid ratio of 1/15 (wt./vol.), and the resulting mixture was maintained at 170 °C for 120 min. Thereafter, the reactor was cooled to room temperature and the reacted mixture was filtered through filter paper with a pore size of 30–50 μm. Then, the filtered residue was washed repeatedly with anhydrous ethanol. The filtrate obtained from the experiment was poured into a pear-shaped flask for rotary evaporation, and ethanol in the filtrate was recovered by evaporation at 55 °C. The filtrate remaining after rotary evaporation was poured into a beaker and deionized water was added to make up the volume to 1000 mL. Then, the pH of the mixture was adjusted between 1 and 2 using concentrated sulfuric acid and the mixture was left undisturbed for 12 h to allow the precipitation of lignin. Acid precipitation is a common method for isolating lignin from an acidic mixture. Lignin dissolved in a basic solution forms a stable colloidal structure owing to the negative charge on its surface. Upon the addition of an acid, H^+^ neutralizes the charge on the colloid surface, causing the colloids to aggregate, leading to the precipitation of lignin (Pang et al. 2021). Therefore, lignin can be separated via its precipitation from the extract by decreasing the pH of the extracted mixture. After the complete precipitation of lignin, the clear supernatant was removed by decantation and the remaining lignin suspension was vacuum-filtered using a microporous membrane with a pore size of 0.45 μm to recover lignin. The collected lignin was washed with deionized water and dried in a vacuum oven at 50 °C. The average extraction yield of lignin was calculated as follows:

1 $$Y \, = \frac{{m_{1} }}{{m_{2} }} \times 100\% ,$$

where *Y* is the extraction yield of lignin, *m*_1_ is the amount (g) of the oven-dried lignin extracted using the organic solvent, and *m*_2_ is the amount (g) of oven-dried Klason lignin.

#### Single-factor experiment of extraction conditions

During the extraction of lignin, the amount of lignin extracted depends on many factors. After a large number of preliminary experiments, four factors including the reaction time, ethanol concentration in the extraction solution, reaction temperature, and solid/liquid ratio were identified to be significantly important factors. The effects of the stirring rate and sulfuric acid content on the extraction yield of lignin were relatively small. In this study, the effects of the extraction time (60, 90, 120, and 150 min), ethanol volume fraction (60, 70, 80, and 90% v/v), solid/liquid ratio (1/10, 1/15, 1/20, and 1/25 g/mL), and reaction temperature (130, 150, 170, and 190 °C) on lignin extraction were investigated while maintaining the other conditions constant.

#### Experimental design

The RSM allows the majorization of the conditions of lignin extraction from palm fibers with minimal number of experiments. A Box–Behnken design with four variables and three levels (− 1, 0, + 1) shown in Table 1 was applied to study the effect of the reaction time (90–120–150 min; *X*_1_), extraction temperature (150–170–190 ℃; *X*_2_), ethanol concentration in the extraction solution (60–70–80% in volume; *X*_3_), and solid/liquid ratio (1/10–1/15–1/20 g/mL; *X*_4_) on the yield of lignin extracted from palm fiber. With the same number of factors, the Box–Behnken scheme requires fewer experiments than the central composite scheme, allows the assessment of nonlinear effects of factors, and is suitable for trials in which all factors are evaluated. The design was implemented for a total of 29 lignin extraction experiments with two replications. The complete experimental design and the response results obtained from the experiments are shown in Table 2.

**Table 1:** Horizontal coding table of the influencing factors in the Box–Behnken design

Variables	Factors	Coded levels		
		− 1	0	+ 1
Reaction time (min)	*X*_1_	90	120	150
Extraction temperature (°C)	*X*_2_	150	170	190
Ethanol solution concentration (% v/v)	*X*_3_	60	70	80
Solid–liquid ratio (g/mL)	*X*_4_	1/10	1/15	1/20

**Table 2:** Experimental results of the RSM

Run	Variables				Response
	Reaction time, *X*_1_, min	Extraction temperature, *X*_2_, ℃	Ethanol concentration, *X*_3_, %	Solid/liquid ratio, *X*_4_, g/mL	Extraction yield, *Y*, %
1	120 (0)	190 (+ 1)	70 (0)	1:20 (+ 1)	43.1
2	120 (0)	190 (+ 1)	60 (− 1)	1:15 (0)	42.6
3	150 (+ 1)	150 (1)	70 (0)	1:15 (0)	23.1
4	120 (0)	150 (− 1)	60 (− 1)	1:15 (0)	28.8
5	90 (− 1)	150 (− 1)	70 (0)	1:15 (0)	42.5
6	150 (+ 1)	170 (0)	80 (+ 1)	1:15 (0)	41.6
7	120 (0)	170 (0)	70(0)	1:15 (0)	55.2
8	120 (0)	190 (+ 1)	70 (0)	1:10 (− 1)	37.8
9	90 (− 1)	170 (0)	70 (0)	1:10 (− 1)	32.3
10	120 (0)	170 (0)	60 (− 1)	1:10 (− 1)	35.6
11	90 (− 1)	170 (0)	60 (− 1)	1:15 (0)	28.3
12	120 (0)	150 (− 1)	80 (+ 1)	1:15 (0)	35.2
13	120 (0)	170 (0)	70 (0)	1:15 (0)	54.8
14	120 (0)	170 (0)	60 (− 1)	1:20 (+ 1)	31.2
15	120 (0)	170 (0)	70 (0)	1:15 (0)	54.2
16	120 (0)	170 (0)	70 (0)	1:15 (0)	54.0
17	120 (0)	150 (− 1)	70 (0)	1:20 (+ 1)	38.2
18	120 (0)	170 (0)	80 (+ 1)	1:10 (− 1)	45.5
19	90 (− 1)	190 (+ 1)	70 (0)	1:15 (0)	43.3
20	150 (+ 1)	190 (+ 1)	70 (0)	1:15 (0)	48.7
21	90 (− 1)	170 (0)	70 (0)	1:20 (+ 1)	46.0
22	90 (− 1)	170 (0)	80 (+ 1)	1:15 (0)	40.3
23	150 (+ 1)	170 (0)	70 (0)	1:20 (+ 1)	30.2
24	120 (0)	170 (0)	80 (+ 1)	1:20 (+ 1)	44.1
25	120 (0)	150 (− 1)	70 (0)	1:10 (− 1)	30.7
26	120 (0)	190 (+ 1)	80 (+ 1)	1:15 (0)	44.1
27	12 0(0)	170 (0)	70 (0)	1:15 (0)	55.8
28	150 (+ 1)	170 (0)	60 (− 1)	1:15 (0)	37.8
29	150 (+ 1)	170 (0)	70 (0)	1:10 (− 1)	38.9

In order to optimize the lignin extraction process, this study investigated the effects of each factor on the amount of extracted lignin based on a large number of single-factor experiments with the reaction time, extraction temperature, ethanol concentration in the extraction solvent, and solid/liquid ratio as the main factors, corresponding to four independent variables, *X*_1_, *X*_2_, *X*_3_, and *X*_4_, respectively. The Design-Expert software was used for the statistical analysis of the data and for optimizing the four factors to determine the optimal extraction parameters. The yield of the extracted lignin was analyzed using the multiple regression model shown below:

2 $$\begin{aligned} Y = & \beta_{0} + \beta_{1} X_{1} + \beta_{2} X_{2} + \beta_{3} X_{3} + \beta_{4} X_{4} + \beta_{11} X_{1}^{2} + \beta_{22} X_{2}^{2} + \beta_{33} X_{3}^{2} + \beta_{44} X_{4}^{2} \\ & + \beta_{12} X_{1} X_{2} + \beta_{13} X_{1} X_{3} + \beta_{14} X_{1} X_{4} + \beta_{23} X_{2} X_{3} + \beta_{24} X_{2} X_{4} + \beta_{34} X_{3} X_{4} , \\ \end{aligned}$$

where *Y* is the response value, $\beta_{0}$, $\beta_{1}$, $\beta_{2}$, $\beta_{3}$, $\beta_{4}$, $\beta_{11}$, $\beta_{22}$, $\beta_{33}$, $\beta_{44}$, $\beta_{12}$, $\beta_{13}$, $\beta_{14}$, $\beta_{23}$, $\beta_{24}$, and $\beta_{34}$ are the regression coefficients, and $X_{1}$, $X_{2}$, $X_{3}$ and $X_{4}$ are the independent variables for the reaction time, extraction temperature, ethanol solution concentration, and solid/liquid ratio, respectively. The fit of the model is indicated by the regression coefficient (*R*^2^). The closer the *R*^2^ value is to 1, the better the model fits, and the ANOVA results are used to analyze the significance of the effect of each factor on the model.

### Characterization of the lignin extract

#### Thermo-gravimetric (TG) analysis

The TG weight of the extracted lignin was measured using a TG analyzer (TGA/DSC3+, Mettler Toledo, Switzerland). The test was performed by weighing ~ 8 mg of the extracted lignin in a crucible and heating it from 35 to 800 ℃ under nitrogen; the heating rate was 10 ℃/min and the nitrogen flow rate was 20 mL/min.

#### Gel permeation chromatography (GPC)

The molecular weight of lignin was determined using a high-performance liquid chromatography system (LC-20AD) using a refractive index detector (RID-20A). The measurements were carried out at a flow rate of 1 mL/min using polystyrene as the standard and tetrahydrofuran (THF) as the mobile phase.

#### SEM analysis

The morphologies and structures of the palm fiber and lignin extracted from it were characterized using scanning electron microscopy (SEM, Zeiss Sigma 500, Germany). An appropriate amount of the sample was placed on conductive gel for photographic observation at an operating voltage of 10 kV.

#### Determination of the structure

Fourier-transform infrared (FTIR) spectra of the extracted lignin samples were recorded on Perkin Elmer spectrum 100. The samples were pressed into pellets by grinding with potassium bromide, and the spectra were obtained in the wavenumber range of 4000–400 cm^−1^. The number of scans was set to four and the resolution was 4 cm^−1^.

#### 1H-NMR analysis

The ^1^H-NMR spectra of the extracted lignin samples were acquired using an NMR spectrometer (WNMR-I 400 MHz) using DMSO-d_6_ as the solvent. The detection resonance frequency of ^1^H was 400.17 MHz, the sampling time was 0.2 s, the spectral width was 5102 MHz, and the number of scans was 16. The ^1^H-NMR spectra were analyzed using MestReNova software.

#### N_2_ adsorption/desorption isotherm analysis

The nitrogen adsorption/desorption isotherms of the adsorbents were recorded on an automated gas sorption analyzer (Autosorb-iQ, Anton-Paar, Austria). First, the dried samples were loaded into the test port. The degassing temperature was set to 180 ℃ and the degassing time was 10 h. The specific surface area, pore size distribution, and pore volume of the two adsorbents were calculated from the isotherms.

### Preparation of lignin-based activated carbon fiber

Ethanol-extracted lignin samples were mixed with PVA (10 wt%) at a weight ratio of 50:50 and dissolved in DMF to prepare a 15 wt% spinning solution (total mass: 10 g). Subsequently, the lignin solution prepared for electrostatic spinning was transferred into a 10-mL medical syringe attached with a 22 gauge needle and spun into fibers using an electrostatic spinning machine. The injection speed of the spinning solution was 0.5 mL/h. The receiving distance was 15 cm. An electronic voltage of 16 kV was applied between the needle tip (14 kV) and cylindrical collector (− 2 kV). The fiber membrane was thermostabilized in a tube furnace in air from room temperature to 180 ℃ at a heating rate of 1 ℃/min for 2 h. Thereafter, the thermostabilized fiber membrane was carbonized in a tube furnace under nitrogen flow (50 mL/min). The temperature was increased from room temperature to 800 ℃ at a heating rate of 10 ℃/min and held there for 1 h. The carbonized fibers were washed with deionized water and dried at 110 ℃; these are referred to as LCFs. In another experiment, the thermostabilized membrane and KOH powder were completely mixed at a weight ratio of 1:3 and then calcined at 800 ℃ according to the aforementioned carbonization conditions. The activated fibers were washed with deionized water and dried. These fibers are referred to as lignin-based activated fibers (LACFs).

## Results and discussion

### Palm fiber chemical constitution

The main components of the palm fiber used in this study are listed in Table 3; the results are in good agreement with those reported earlier (Li et al. 2020). Similar to other natural fibers, the palm fibers are mainly composed of cellulose, hemicellulose, and lignin. The cellulose content of the palm fiber (26.1%) is relatively low compared with those of the other members of the palm family, such as oil palm (36.8%) (Rashid et al. 2018) and sugar palm (43.8%) (Ilyas et al. 2018). However, compared with oil palm (26.0%) (Mahmood et al. 2019), the palm fiber (34.6%) has a relatively high lignin content. According to Rashid et al. (2018), a higher lignin content in the biomass network contributes to superior recalcitrant characteristics of the biomass during its dissolution and fractionation. Therefore, a higher extraction temperature and longer reaction time might be required in the lignin extraction process.

**Table 3:** Composition of the palm fiber (dry mass wt. %)

Chemical component	%w/w in crude material	Testing method
Lipid wax	3.6 ± 0.12	GB/T 5889-86 (benzene:ethanol, 2:1 in volume)
Water-soluble substance	3.8 ± 0.16	GB/T 5889-86 (distilled water)
Pectin	3.4 ± 0.14	GB/T 5889-86 (5 g/L ammonium oxalate)
Hemicellulose	28.5 ± 0.43	GB/T 5889-86 (20 g/L NaOH)
Klason lignin	34.6 ± 0.21	NREL10.1.6 (72% H_2_SO_4_)
Cellulose	26.1 ± 0.36	GB/T 5889-86

### Influence of experimental conditions on lignin extraction

During the extraction of lignin from palm fibers, the extraction yield of lignin is likely to be affected by many factors. After a large number of preliminary experiments, the following four factors were identified as the most important ones: the reaction time, extraction temperature, ethanol content in the extraction solvent, and solid/liquid ratio. To optimize the process, the effect of each parameter on the lignin yield, viz., the reaction time (60, 90, 120, and 150 min), extraction temperature (130, 150, 170, and 190 °C), ethanol concentration (60, 70, 80, and 90% v/v) in the solvent, and solid/liquid ratio (1/10, 1/15, 1/20, and 1/25 g/mL) on the extraction efficiency of lignin using ethanol was investigated.

#### Reaction time

**Fig. 2:**
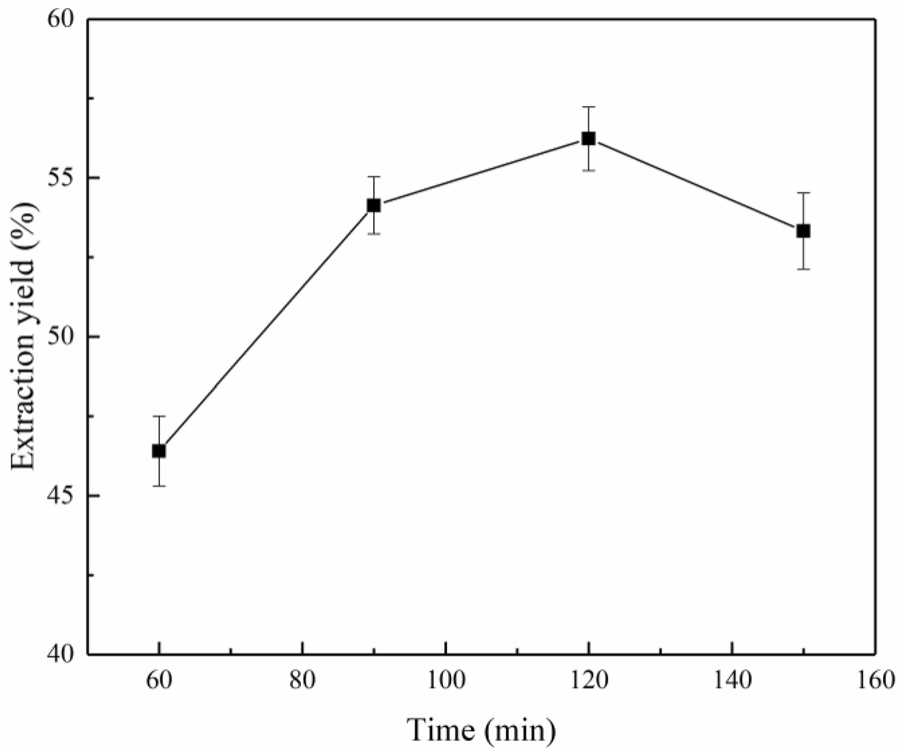
Influence of the reaction time on the lignin extraction yield at the extraction temperature of 170 °C using 70% aqueous ethanol and a solid/liquid ratio of 1/15 g/mL

The reaction time significantly influenced the extraction of lignin from palm fibers. As shown in Fig. 2, when the reaction time was between 60 and 120 min, the lignin yield exhibited an increasing trend. As the reaction time exceeded 120 min, a small decrease in the lignin yield was noted. These results are in complete agreement with the observations made by Ramezani et al. (2019). When the reaction time reached 120 min, the palm fiber lignin dissolved completely. However, when the reaction time was prolonged, some of the extracted lignin decomposed, therefore, the extraction yield decreased when the reaction time exceeded 120 min.

#### Extraction temperature

**Fig. 3:**
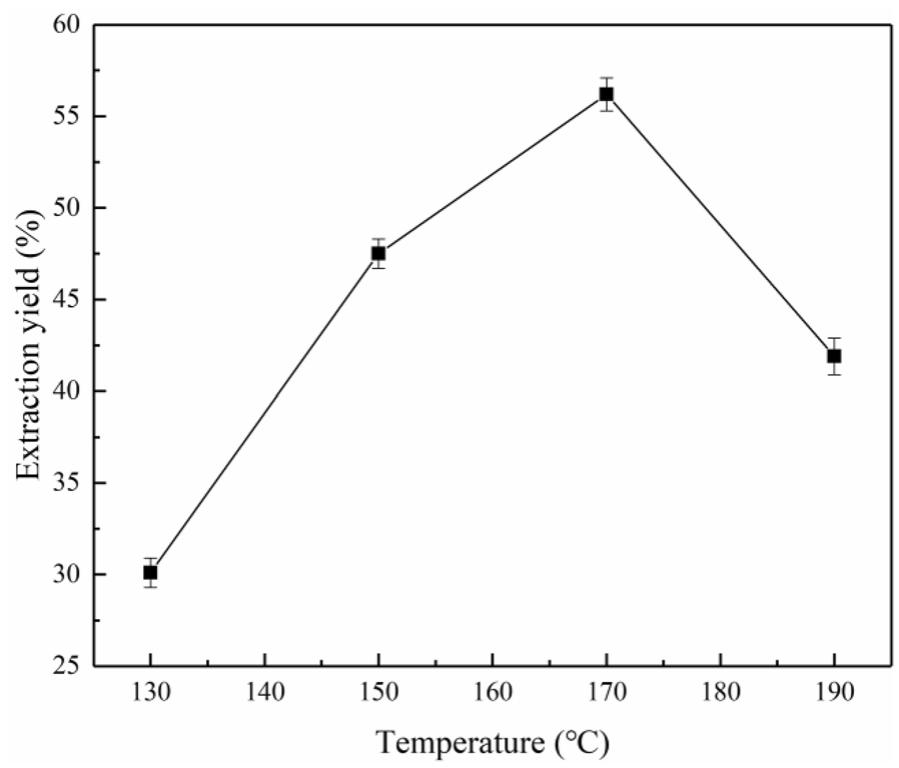
Effect of the extraction temperature on the lignin extraction yield under the extraction conditions of 120 min reaction time using 70% ethanol at 1:15 g/mL solid/liquid ratio

The influence of the extraction temperature on the lignin extraction yield is illustrated in Fig. 3. In the temperature range of 130 to 170 °C, the extraction yield of lignin increased significantly with increasing temperature, and then the yield decreased with a further increase in temperature. This is in good agreement with Zhang et al. (2016), who extracted lignin from corn straw using aqueous ethanol as the solvent and observed that the lignin yield increased with increasing temperature in the range of 100–180 °C. This behavior is most probably due to the increased solubility of lignin with increasing temperature. Above 170 °C, the extraction yield of lignin began to gradually decrease. Higher operating temperatures and prolonged reaction times have a detrimental effect on the extraction of lignin, which may disrupt the fragmentation of the ether bonds in lignin, further leading to the formation of other compounds and the degradation of the extracted lignin (Rashid et al. 2018).

#### Ethanol concentration

**Fig. 4:**
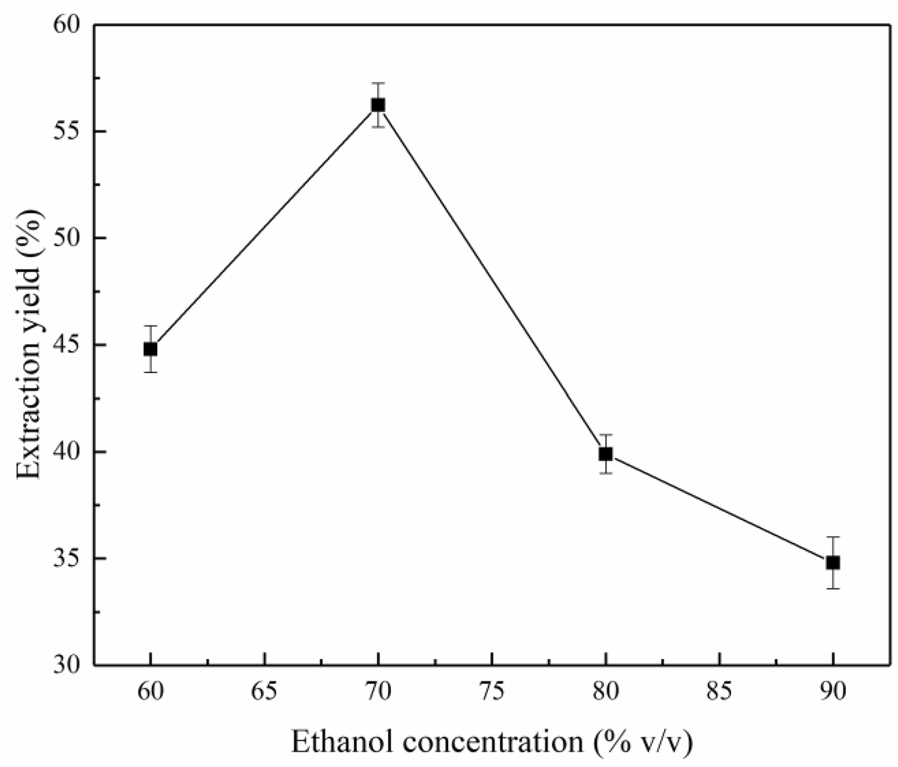
Effect of the ethanol concentration on lignin extraction under the conditions of reaction time of 120 min, extraction temperature of 170 °C and solid/liquid ratio of 1:15 g/mL

The ethanol concentration in the extraction solvent had a notable effect on the lignin extraction yield. As shown in Fig. 4, in the investigated range of ethanol concentration, 60–70%, the extractability of the solvent increased with increasing ethanol volume fraction, and a maximum yield of 56.2% was achieved at 70% ethanol. However, when the volume fraction of ethanol exceeded 70%, the lignin extraction yield decreased significantly. This result is similar to the observation made by Ye et al. (2012), who reported that the highest lignin yields were achieved upon using 65% ethanol in water as the solvent. This may be because the higher the ethanol concentration is, the lower the water content in the reaction system is and the lower the heat capacity of the system is, which is not conducive to the breakage of the ether bonds of lignin, resulting in lower lignin yields. Similar results were obtained earlier in the extraction of lignin from mango seed husks using ethanol-based solvents (Bello and Chimphango 2021).

#### Solid/liquid ratio

**Fig. 5:**
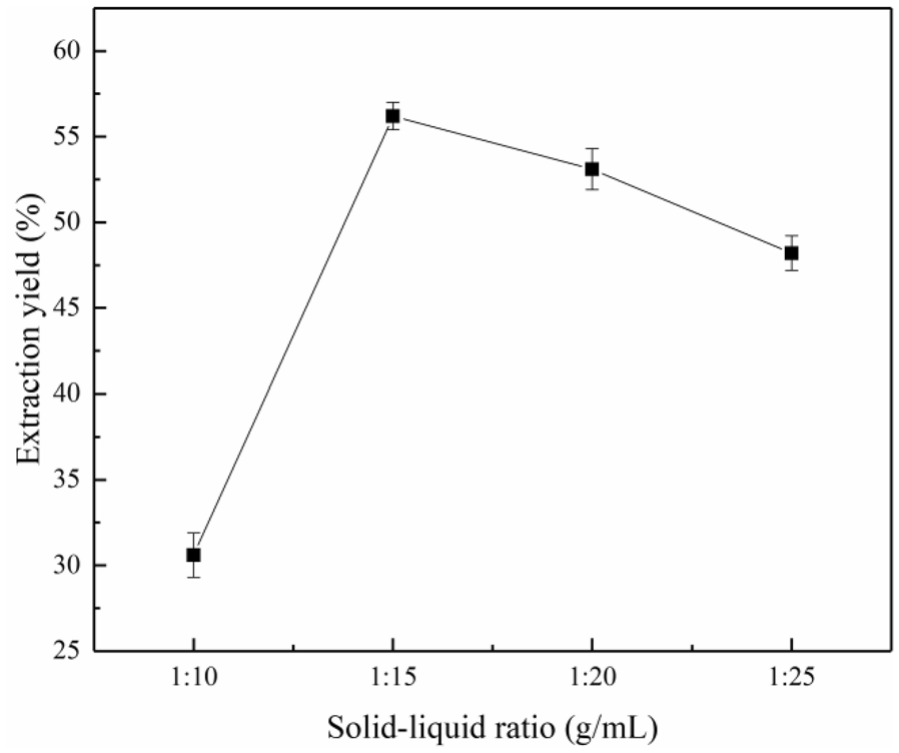
Effect of the solid/liquid ratio on the yield of lignin extracted under the conditions of a reaction time of 120 min, extraction temperature of 170 °C, and ethanol concentration of 70%

The effect of the solid/liquid ratio on the lignin extraction yield is shown in Fig. 5. The lignin extraction yield increased significantly as the solid/liquid ratio increased from 1/10 to 1/15 g/mL, which is very similar to the results reported by Qin et al. (2021). This is because the greater the amount of liquid with respect to the solid biomass is, the greater the dissolution of lignin is. As the solid/liquid ratio was increased above 1/15 g/mL, the lignin yield began to decline at a slow rate. An excess of the solvent caused a decrease in the cohesive energy between the palm fiber and extraction solution, resulting in decreased extraction of lignin (Rashid et al. 2018).

### Optimal extraction conditions of lignin from palm fibers

#### Establishment of the mathematical model and significance test

The experimental data in Table 4 were analyzed and processed using the Design-Expert software, and the regression formula (Eq. 3) of the lignin extraction yield was obtained via regression analyses.

**Table 4:** Analysis of variance for the regression equation of the lignin extraction yield

Source	Sum of squares	Degree of freedom	Mean square	*F*-value	*P* > *F*	
Model	2003.61	14	143.11	9.56	< 0.0001	Significant
*X*_1_	12.81	1	12.81	0.86	0.3705	
*X*_2_	311.10	1	311.10	20.79	0.0004	
*X*_3_	180.19	1	180.19	12.04	0.0038	
*X*_4_	12.00	1	12.00	0.80	0.3857	
*X*_1_ *X*_2_	153.76	1	153.76	10.27	0.0064	
*X*_1_* X*_3_	16.81	1	16.81	1.12	0.3072	
*X*_1_* X*_4_	125.44	1	125.44	8.38	0.0118	
*X*_2_* X*_3_	6.00	1	6.00	0.40	0.5367	
*X*_2_* X*_4_	1.21	1	1.21	0.081	0.7803	
*X*_3_* X*_4_	2.25	1	2.25	0.15	0.7040	
*X*_1_^2^	489.55	1	489.55	32.71	< 0.0001	
*X*_2_^2^	420.34	1	420.34	28.09	0.0001	
*X*_3_^2^	460.41	1	460.41	30.77	< 0.0001	
*X*_4_^2^	481.14	1	481.14	32.15	< 0.0001	Significant
Residual	209.51	14	14.96			
Lack of fit	207.35	10	20.73	38.40	0.0016	
Pure error	2.16	4	0.54			
Cor total	2213.11	28				

3 $$\text{Extraction yield},Y = 54.80-1.03{X}_{1}+ 5.09{X}_{2}+ 3.88{X}_{3}+ 1.00{X}_{4}+ 6.20{X}_{1}{X}_{2}-2.05{X}_{1}{X}_{3}-5.60{X}_{1}{X}_{4}-1.23{X}_{2}{X}_{3}-0.55{X}_{2}{X}_{4}+ 0.75{X}_{3}{X}_{4}-8.69{{X}_{1}}^{2}-8.05{{X}_{2}}^{2}-8.42{{X}_{3}}^{2}-8.61{{X}_{4}}^{2}.$$

The correlation coefficient (*R*^2^) was used to assess the quality of the model. The *R*^2^ value of this model was determined to be 0.9053, which indicates that 90.53% of the fluctuations in the extraction yield of lignin could be accounted by each influencing factor, suggesting that the model is realistic and reliable. The *R*^2^ value of the lignin extraction yield was close to 1, indicating that the predicted values of the model were consistent with the experimental results.

When a multivariate quadratic polynomial is fitted using the RSM, the fitted quadratic model needs to be judged. The results of the ANOVA of the RSM are shown in Table 4, and the *p*-values in the table are mainly used to judge the significance of each variable in the fitted multivariate quadratic polynomial equation. For *p*-value > 0.05, the effect is not significant; for *p*-value between 0.01–0.05, the effect is significant; and for *p*-value < 0.01, the effect is highly significant. As shown in Table 4, the *p*-value of the entire model is < 0.0001, which confirms that the effect of the model is highly significant and the model can accurately reflect the relationship between the response values and each influencing factor. Moreover, the interaction effects of *X*_1_*X*_2_, *X*_1_*X*_4_, *X*_1_^2^, *X*_2_^2^, *X*_3_^2^, and *X*_4_^2^ were significant, and the effects of the remaining interaction terms were not significant.

#### Response surface stereogram analysis

**Fig. 6:**
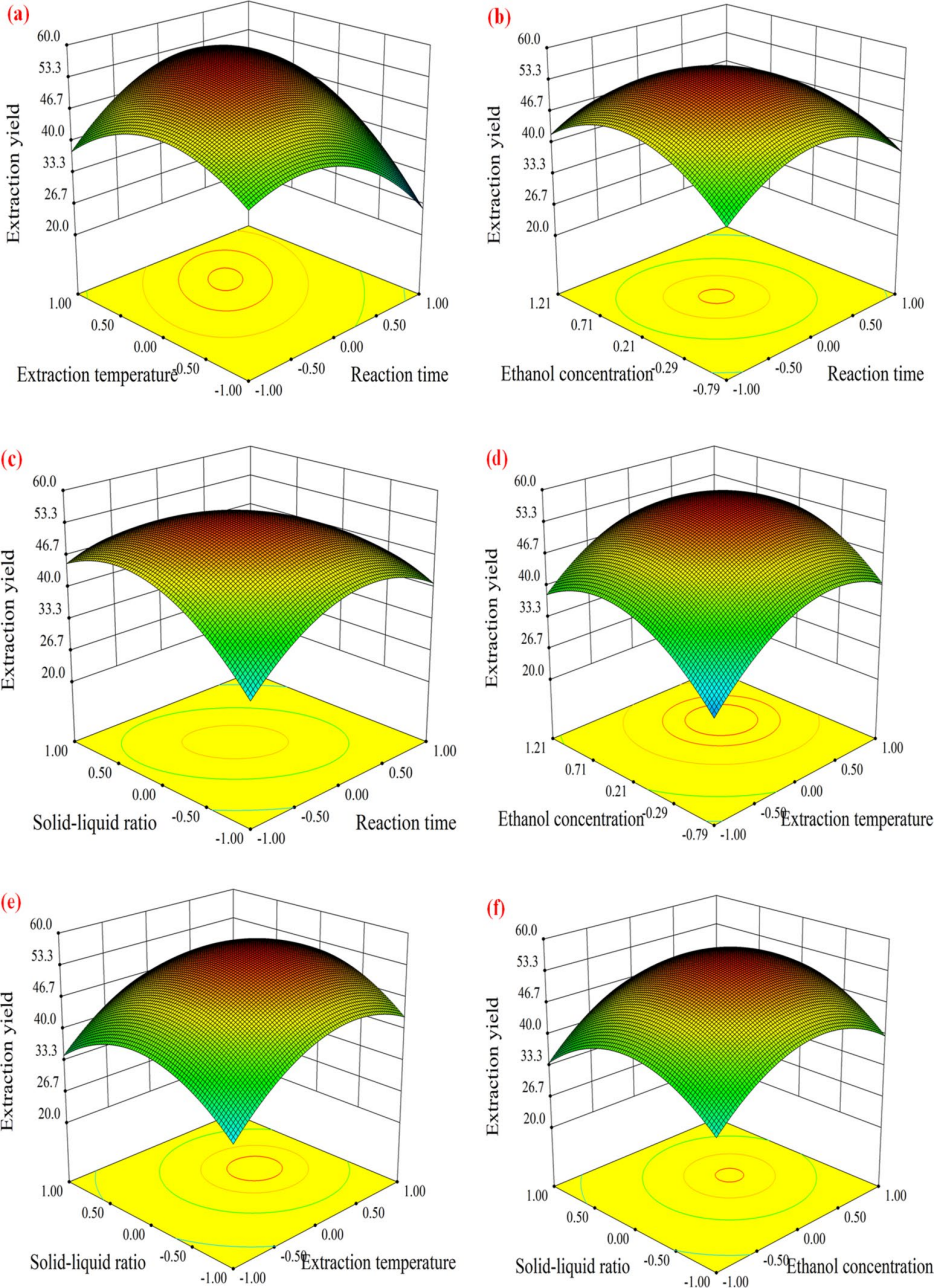
Effects of different parameters on the yield of lignin extraction from palm fibers using ethanol: interaction effects of the **a** extraction time and reaction temperature, **b** extraction time and ethanol concentration, **c** extraction time and solid/liquid ratio, **d** reaction temperature and ethanol concentration, **e** reaction temperature and solid/liquid ratio, and **f** ethanol concentration and solid/liquid ratio

The effects of the interactions of the different variables on the extraction yield of lignin were studied by drawing three-dimensional surfaces, and the optimal impact level of each variable was determined. Surface plots demonstrating the interaction of a given pair of examined factors on the ethanol-based extraction of lignin are presented in Fig. 6a–f.

Figure 6a shows the dependence of the lignin extraction yield on the reaction temperature and extraction time. The lignin extraction yield is low at low reaction temperatures and long reaction times. The lignin extraction yield increases with increasing extraction temperature and time, but a high extraction temperature and prolonged reaction time adversely affect the extraction of lignin. This may disrupt the fragmentation of ether bonds in lignin and in turn lead to the degradation of lignin and the production of other compounds (Zhang et al. 2016).

The ANOVA results indicated a linear effect of the ethanol concentration and extraction temperature on the yield of lignin, and their interaction was also found to have a remarkable effect on the extraction yield of lignin. The response surface plot in Fig. 6b indicates that the extraction yield of lignin increases as the ethanol concentration and extraction temperature increase. When the ethanol concentration is increased above 73%, the extraction yield of lignin decreases (Bello and Chimphango 2021).

Figure 6c reveals the effect of the interaction of the extraction time and solid/liquid ratio on the lignin extraction yield. The plot indicates that a short extraction time (114 min) and low solid/liquid ratio (1/10 g/mL) leads to the lowest lignin extraction yield (23.1%) (Ramezani and Sain 2019)^.^

The influence of the extraction temperature and ethanol concentration on the lignin extraction yield is presented in Fig. 6d. The plot indicates that the lignin extraction yield increases with the increase in the extraction temperature and ethanol concentration, and the lignin extraction yield is maximized when the extraction temperature reaches 176.8 °C and the ethanol concentration reaches 72.1%. However, the extraction yield would decrease significantly when the temperature and concentration increase beyond the above values. This may be because a higher ethanol concentration and reaction temperature promote the esterification reaction of ethanol, leading to lesser catalytic cleavage of the ether bond of lignin and a decrease in the yield of lignin (Rashid et al. 2021).

The effect of the interaction of the reaction temperature and solid/liquid ratio on the lignin extraction yield is presented in Fig. 6e. Both low and high solid/liquid ratios have an adverse effect on the extraction yield of lignin. The maximum yield of lignin is observed at 171 °C corresponding to a solid/liquid ratio of 1/15 g/mL. When the solid/liquid ratio is low, the solvent barely submerges the material, resulting in insufficient contact between the solvent and palm fiber owing to the thermal expansion of the material and solvent vaporization during the heating process, which results in a low yield of lignin. When the solid/liquid ratio is high, the ethanol content is high and the esterification between ethanol and the hemicellulose acid in the palm fiber is accelerated, which causes the loss of lignin by the formation of esters and other byproducts (Minjares-Fuentes et al. 2016).

The interaction of the ethanol concentration and solid/liquid ratio on the lignin extraction yield is presented in Fig. 6f. When the solid/liquid ratio is low, the heat capacity of the reaction system is limited due to the small amount of solvent, and the energy available for lignin cracking and dissolution is also less, resulting in a low lignin yield. When the ethanol concentration is high, the water content in the reaction system is smaller, the heat capacity of the system is smaller, and the energy absorbed is less, which is unfavorable for the breakage of the ether bond of lignin. This results in a decreased yield of lignin (Li et al. 2015; Ratanasumarn and Chitprasert 2020).

Through Design-Expert analyses (Fig. 6), the following optimal values of the four variable were obtained: a reaction time of 111 min, extraction temperature of 174 °C, ethanol concentration of 73%, and solid/liquid ratio of 1:16 g/mL. The model predicted an extraction yield of 55.9%, which was experimentally verified. A lignin extraction yield of 56.2% was achieved experimentally under the optimal conditions derived from modeling; this value is in excellent agreement with the predicted value. Thus, the optimal conditions for the extraction of lignin from palm fibers using ethanol were identified, and the experimental results indicated that the processing parameters optimized by the RSM are reliable.

### Solvent recovery and its effect on extraction yield of lignin

**Fig. 7:**
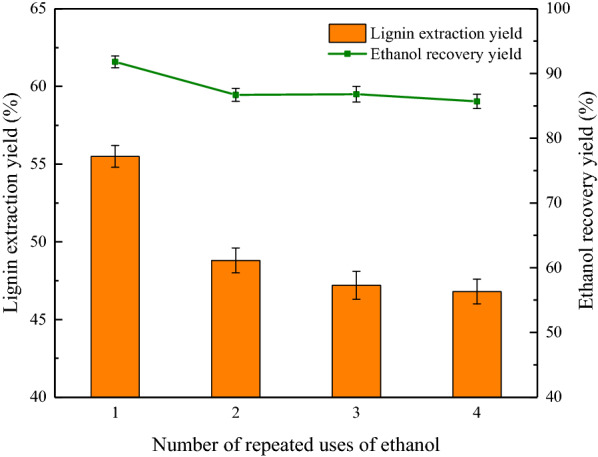
Influence of solvent recycling on the lignin extraction rate and efficiency of ethanol recovery

In order to develop an ecofriendly and industrially viable technology for lignin extraction from biomass, the solvent should be recovered without the loss of its extraction efficiency. To accomplish this, after extraction, the lignin solution was subjected to rotary vacuum evaporation at 55 °C under low pressure (0.06 MPa), and more than 90% of ethanol was recovered. The recovered ethanol was cycled in four consecutive extraction experiments; that is, the ethanol recovered in each extraction cycle was used in the subsequent one. The lignin extraction efficiencies in the four consecutive cycles were 55.5, 48.8, 47.2, and 46.8% with the ethanol recovery being 91.8, 86.7, 86.8, and 85.7%, respectively. As shown in Fig. 7, both the lignin extraction efficiency and the solvent recovery efficiency decreased significantly after the first extraction cycle. After four cycles, the lignin extraction efficiency and solvent recovery stabilized at ~ 85% and ~ 46%, respectively, suggesting that ethanol can be a viable and recoverable solvent in lignin extraction from biomass (Ramezani and Sain 2019).

### Characterization of the lignin extract

#### TGA

**Fig. 8:**
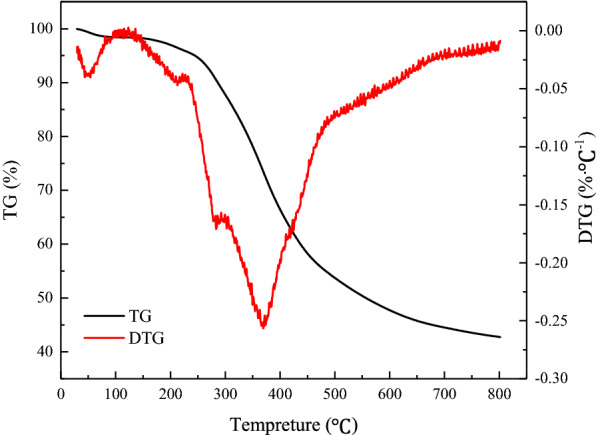
Thermo-gravimetric analysis of lignin

The thermal stability of the extracted lignin was evaluated by TGA. The TG and DTG curves in Fig. 8 show the mass loss from lignin and the corresponding rate as functions of temperature. The TG curve of the lignin sample can be roughly divided into three parts. During the first stage of mass loss in the temperature range of 35–150 °C, the degradation of lignin was slow, and the main reason for mass loss is the volatilization of free water and some small molecules (Shi et al. 2019). In the second stage of mass loss between 150 and 480 °C, the cleavage of the bonds linking the lignin structural units occurs, resulting in the production of phenolics and some gases (Shi et al. 2019). Finally, the mass loss rate of lignin increased in the third stage between 480 and 800 °C, during which the aromatic rings in the lignin structure started to decompose, resulting in further mass loss (Teh et al. 2021). At 800 °C, the residual mass of lignin was still as high as 42%. According to a previous study (Abdollahi et al. 2019), the Kraft lignin completely degraded at the pyrolysis temperature of 700 °C. In comparison, the lignin extracted from palm fibers using ethanol in the present study has excellent thermal stability.

#### Molecular weight analysis

**Fig. 9:**
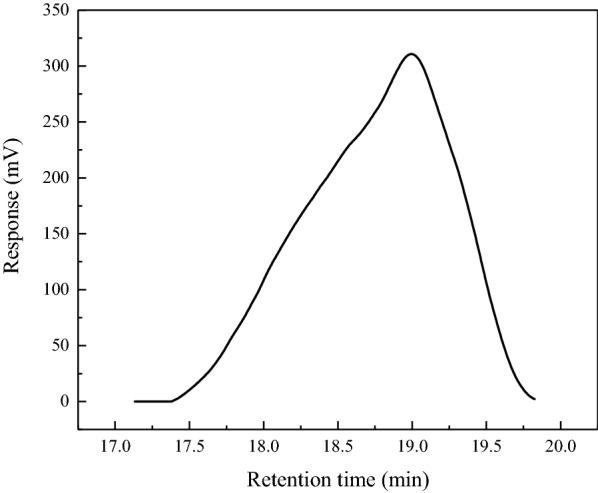
Molecular weight distribution of the palm fiber lignin

The extracted lignin was found to have a typical molecular weight distribution, as shown in Fig. 9. Further, as shown in Table 5, the weight-average molecular weight (Mw) of the palm fiber lignin extracted using ethanol is 864 Da, which is comparable to the Mw (1195 Da) of lignin extracted using ethanol by Meng et al. (2019). However, some differences could be noted between the two results because of the distinct acid precipitation behaviors of lignin with different molecular weight distributions. At a pH of 1–2 used in the present study, the low molecular-weight lignin fraction precipitates more predominantly than the high molecular-weight one (Pang et al. 2021). The ethanol-extracted lignin obtained in this study has a relatively low polydispersity index (PDI) of 1.34, which indicates that it has a lower molecular weight distribution and higher homogeneity than that reported earlier (PDI < 1.92) (Rashid et al. 2016). In biorefinery processes, the dispersion coefficient of lignin is an important parameter, and a low dispersion coefficient implies a better biochemical stability with a wider range of prospective applications. The lignin extracted from palm fibers using ethanol has a low PDI, which is another advantage in its subsequent processing.

**Table 5:** Molecular weight analysis of the lignin extracted from palm fibers

Molecular weight	Lignin (g/mol)
Mn	645
Mw	864
PDI (Mw/Mn)	1.34

#### SEM analysis

**Fig. 10:**
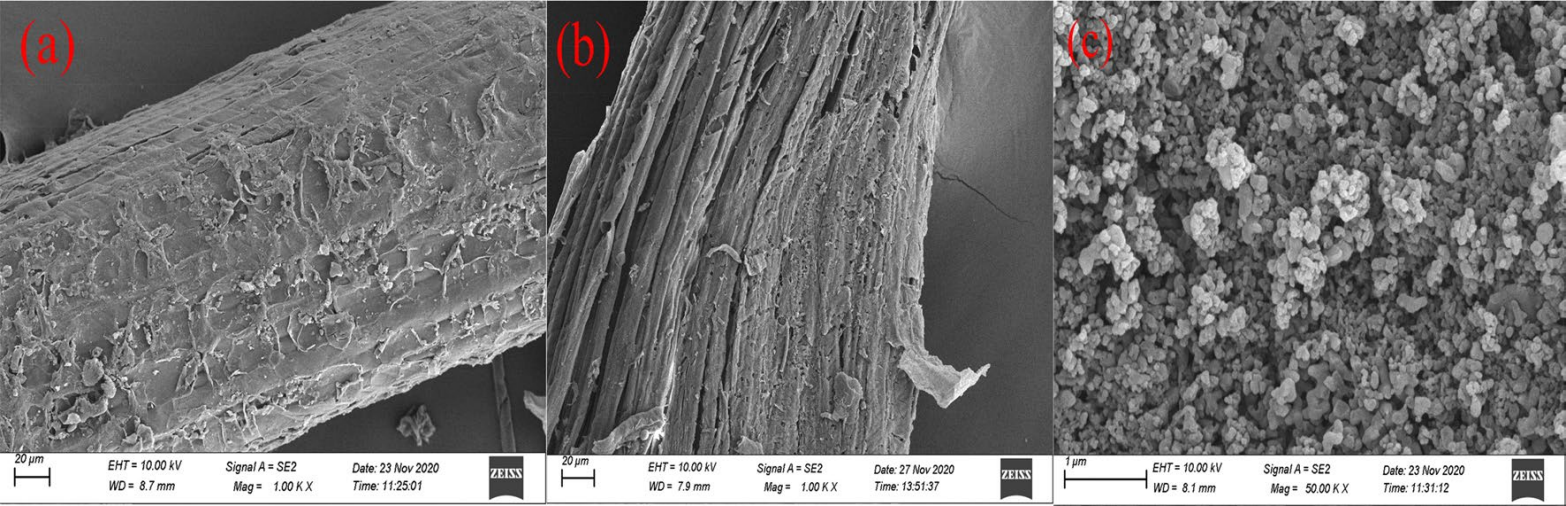
SEM images of the original palm fiber (**a**), palm fiber treated with ethanol for lignin extraction (**b**), and the extracted lignin (**c**)

The SEM images of the original palm fiber and the processed fiber (after lignin extraction) are shown in Fig. 10. The raw palm fiber has a rough surface, dense structure, and tightly bound fibers with an orderly arrangement. During the high-temperature hydrothermal treatment of the palm fiber using ethanol, the lignin in its structure was effectively cleaved and extracted, which rendered the fiber loose and created gaps in the structure (Abdul Khalil et al. 2011). The SEM image of the extracted lignin is shown in Fig. 10c. It mainly presents roughly spherical granules of lignin with an irregular morphology, and a large amount of lignin aggregates. The observed morphology is similar to the micromorphology of lignin extracted from wheat straw by Li et al. (2019). In addition, the spherical lignin particles have a loose hydrophilic network structure, which provides the advantages of a large surface area, permeability, and good hydraulic properties. These features render it a suitable precursor for preparing an adsorbent (Huang et al. 2017).

#### FTIR spectral analysis

**Fig. 11:**
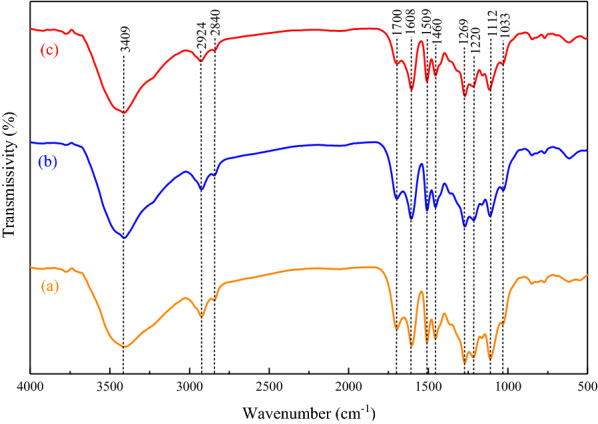
Infrared spectra of the lignin samples extracted from palm fibers under different processing conditions: **a** optimal conditions; **b** conditions of 90 min, 170 °C, 70% ethanol, 1/20 g/mL solid/liquid ratio; and **c** conditions of 90 min, 190 °C, 70% ethanol, 1:15 g/mL solid/liquid ratio

The FTIR spectra of three lignin samples extracted under different conditions are shown in Fig. 11. There is a characteristic absorption band at 3409 cm^−1^, which is the stretching vibration of the alcoholic hydroxyl group on the benzene ring. The hydroxyl group is one of the important functional groups that significantly influences the physical and chemical properties of lignin (Ramezani and Sain 2018). The peaks at 2924 and 2840 cm^−1^ are derived from the C–H stretching vibrations of the methyl and methylene groups, respectively. The peak at 1700 cm^−1^ is due to the C=O stretching vibration in nonconjugated ketones, carbonyl groups, and esters. This peak shifts to a lower wavenumber as hydrogen bonds are easily formed between carbonyl and hydroxyl groups, resulting in decreased vibrational frequency of the carbonyl groups (Bello and Chimphango 2021). Further, the peaks at 1608 and 1509 cm^−1^ are due to aromatic C=C bending vibrations, and can be attributed to the aromatic skeleton of lignin (Florian et al. 2019). The peak at 1460 cm^−1^ represents the C–H bending vibration. In the FTIR spectrum of lignin, the signal of the syringyl (S) unit appears at 1320 cm^−1^, while that of the guaiacyl unit (G) appears at 1269 cm^−1^ (Ji et al. 2022). The bands between 1300 and 1000 cm^−1^ can be attributed to the C–O stretching vibration of the ether or ester linkages. In Table 6, the FTIR spectral peaks of the lignin extracted from palm fibers are compared with the spectral data obtained for lignin extracted from palm mesocarp fibers by Rashid et al. (2018). The IR absorption peaks obtained for the lignin extracted in this study reveal a typical lignin chemical structure, and no significant difference was observed between the lignin structures extracted under different conditions (Xu et al. 2013; Mamilla et al. 2019).

**Table 6:** FTIR band assignments for the lignin extracted from palm fibers

Palm mesocarp fiber lignin (Rashid et al. 2018) Band (cm^−1^)	Palm fiber this work Band (cm^−1^)	Peak assignment
3419	3409	O–H stretching
2935	2924	C–H stretching
2850	2840	C–H stretching (O–CH_3_)
1716	1700	C–H vibration of aromatic ring
1651	1608	CH vibration of aromatic ring
1554		C=O stretching
1512	1509	Aromatic skeletal vibration
1459	1460	Asymmetric bending vibrations of methyl and methylene groups
1425		C–H in-plane bending vibration
1372		C–H bending vibration in methyl
1325	1320	Vibrations of syringyl units
1269	1269	Carbonyl stretching vibration of guaiacyl units
1034	1033	C–O deformations of aliphatic groups

#### ^1^H-NMR analysis

**Fig. 12:**
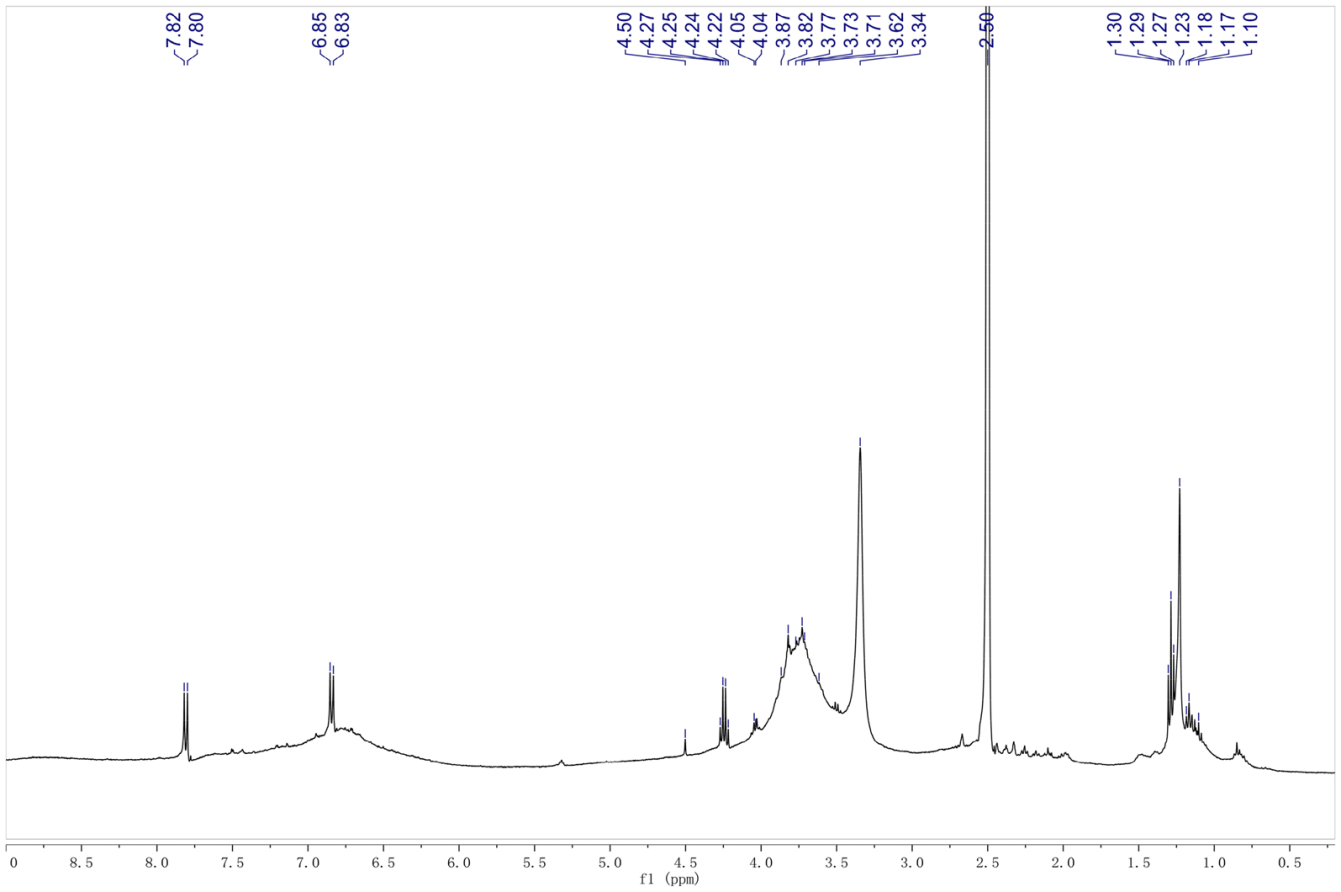
^1^H-NMR spectrum of the lignin extracted from palm fibers using ethanol

The analysis of the ^1^H-NMR signal intensities is an appropriate method for assessing the structural purity of the extracted lignin. The ^1^H-NMR spectrum of the ethanol-extracted lignin is shown in Fig. 12, and the NMR peak positions and the corresponding assignments of the protons in the lignin products are listed in Table 7. The signals were assigned based on previous reports (Rashid et al. 2016). The peak at ~ 1.2 ppm in the ^1^H-NMR spectrum corresponds to the hydrogen atoms in aliphatic groups. The sharp peak at 2.5 ppm is due to DMSO, the solvent used for dissolving the extracted lignin. The peak at ~ 3.35 ppm corresponds to the protons of the methoxy group in the benzene ring of lignin. The peaks at 3.6–3.8 are assigned to the Hα, Hβ, and hydroxyl groups in the side chain of the benzene ring (Gunasekaran et al. 2020). The peaks at 4.2–4.5 ppm represent Hα in the β-β and β-O-4 linkage structures, but the peak at 4.5 ppm was not clearly observable in the spectrum, indicating that the β-O-4 functional group might have decomposed substantially during the lignin dissolution process (Wang et al. 2020). The peaks at 6.8–7.8 ppm represent the aromatic ring protons in the guaiacyl (G) and syringyl (S) units (Coral Medina et al. 2015). Based on the analyses, it can be concluded that the ^1^H-NMR spectrum of the palm fiber lignin is similar to those reported earlier. This result also suggests that the lignin structure was not significantly damaged during the separation process, indicating the successful extraction of lignin from palm fibers using ethanol.

**Table 7:** Major ^1^H-NMR signals of lignin and the corresponding assignments

Proton shift assignments of lignin	
Signal (ppm)	Attribution
6.8–7.8	Aromatic H in G and S units
4.2–4.5	Hα in β-β and β-O-4 structures
3.6–3.8	H in the side chains of benzene rings
3.35	Methoxy H
2.5	DMSO (solvent) peak
1.2–1.3	H in aliphatic groups

### Water vapor adsorption capacity of carbon fibers derived from lignin

**Fig. 13:**
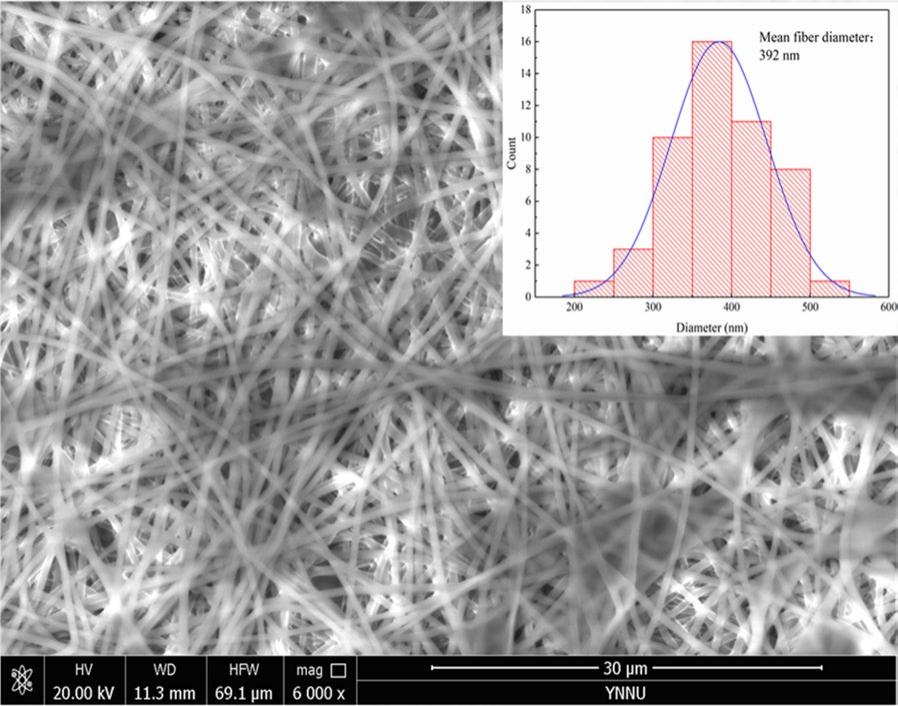
SEM image and diameter distribution of raw lignin fibers

**Fig. 14:**
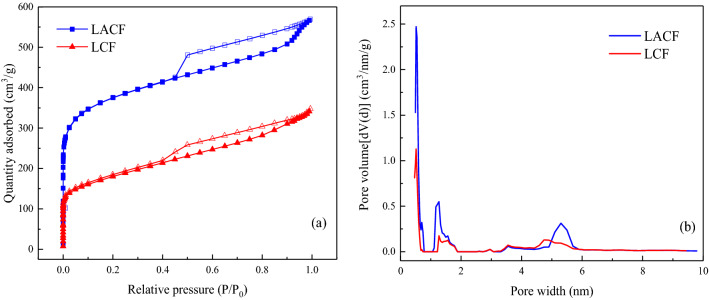
**a** N_2_ adsorption/desorption isotherms recorded at 77 K and **b** DFT pore size distribution of the LCF and LACF

**Fig. 15:**
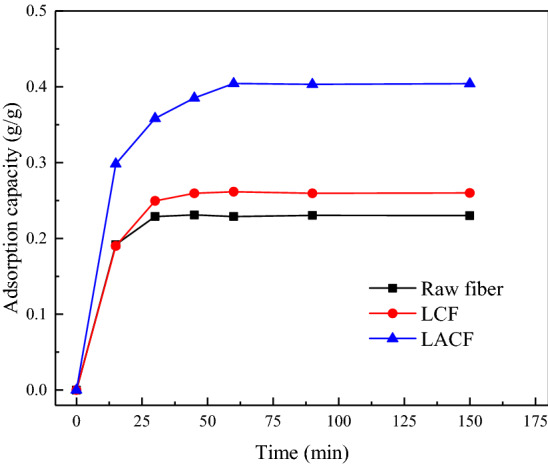
Kinetics of water vapor adsorption to the raw fiber, LCF, and LACF

Figure 13 shows a SEM image of the raw electrospun fibers of the lignin/PVA blend. The raw lignin/PVA fibers had a uniform and smooth morphology; no beads were formed and the average diameter was 392 nm (Santos et al. 2014). The results suggest that the ethanol-extracted lignin can be successfully electrospun into nanofibers without further purification or refinement. In this study, the raw fibers of the lignin/PVA blend were further calcined and activated for assessing the potential of the resultant carbon fibers as dehumidifying agents. The specific surface area and pore size distribution of an adsorbent are the most important factors that determine the adsorption performance. Figure 14a, b shows the N_2_ adsorption/desorption isotherms obtained at 77 K and the DFT pore size distribution of the LCF and LACF samples. According to the IUPAC classification of isotherms, the N_2_ adsorption/desorption isotherms of the LCF and LACF are of type I. In the initial stage of adsorption (*P*/*P*_0_ < 0.1), the amount of nitrogen adsorbed by the carbon fibers increased sharply, possibly due to nitrogen adsorption to the large number of micropores present in the carbon fibers. A significant hysteresis loop was observed between the relative pressure of 0.4 and 0.9, which indicated the presence of a certain amount of mesoporous structures in the carbon fibers (Zahid et al. 2021). Figure 14b reveals that most of the pore sizes of the LCF and LACF were distributed in the microporous domain below 2 nm, and only a small fraction was concentrated in the mesoporous domain at approximately 5 nm. The specific surface area and pore volume of the LACF were determined to be 1375 m^2^/g and 0.881 cm^3^/g, respectively, while those of the LCF were 637 m^2^/g and 0.598 cm^3^/g, respectively. The results indicate significant increases in the two parameters after the activation step. Furthermore, the specific surface area of LACF was higher than that of ACFK (1147 m^2^/g) prepared from alkaline lignin by Song et al. (2019). Figure 15 shows the kinetics of water vapor adsorption onto the raw fiber, LCF, and LACF at 25 °C and 70% relative humidity. After the water vapor adsorption time exceeded 60 min, the amount of water vapor adsorbed by the raw fiber, LCF, and LACF barely changed with time, indicating that all samples have very fast water vapor adsorption rates. The water vapor adsorption capacity of the LACF was significantly higher at 0.40 g/g, compared to those of the other two. Thus, LACF had better adsorption capacity for water vapor under the same temperature and humidity conditions, and its water vapor adsorption capacity is also higher than that of the commercial pitch-based activated carbon fiber (0.35 g/g) (Velasco et al. 2021).

## Conclusion

In this study, the RSM was used to successfully determine the optimal conditions for the extraction of lignin from palm fibers using ethanol as a cosolvent. The prediction profile and regression equation indicated that the optimal conditions for lignin extraction are a reaction time of 111 min, extraction temperature of 174 °C, ethanol concentration of 73%, and solid/liquid ratio of 1/16 g/mL. Under the optimum conditions, the lignin extraction yield was 56.2%, agreeing well with the predicted yield. In addition, the recovery of ethanol from the extraction solvent was as high as 91.8%, and the lignin extraction yield with the use of recovered ethanol was satisfactory even after four cycles of extraction, which significantly decreases the extraction cost. Furthermore, the extracted lignin contained abundant functional groups and exhibited good thermal stability. Moreover, it also had a lower molecular weight (864 Da) and good polydispersity (1.34), indicating that a relatively homogeneous lignin fraction was obtained. The extracted lignin could be successfully electrospun by combining with PVA to prepare LACFs. When used as a dehumidifying agent, the water vapor adsorption capacity of the LACF reached 0.40 g/g, which is comparable to that of the commercial asphalt-based activated carbon fiber. It can be concluded that the lignin extracted from palm fibers has a uniform molecular weight and excellent characteristics of raw lignin, which render it an excellent precursor that can be widely used in the preparation of carbon materials, biological composites, and resins.

## Data Availability

The data supporting the conclusions are included in the main manuscript.

## Abbreviations

FTIR: Fourier-transform infrared
TGA: Thermo-gravimetric analysis
SEM: Scanning electron microscopy
^1^H-NMR: 1H nuclear magnetic resonance
RSM: Response surface methodology
GPC: Gel permeation chromatography
DMSO-d_6_: Dimethyl sulfoxide-d6
PVA: Polyvinyl alcohol
DMF: *N, N*-Dimethylformamide
LCF: Lignin-based carbon fiber
LACF: Lignin-based activated carbon fiber
ANOVA: Analysis of variance
PDI: Polydispersity index
